# Inequalities in life expectancy in Australia according to education level: a whole-of-population record linkage study

**DOI:** 10.1186/s12939-021-01513-3

**Published:** 2021-08-03

**Authors:** J Welsh, K Bishop, H Booth, D Butler, M Gourley, HD Law, E Banks, V Canudas-Romo, RJ Korda

**Affiliations:** 1 Research School of Population Health, Australian National University, Building 62, Mills Rd, ACT 2601 Acton, Australia; 2 School of Demography, Australian National University, Acton, Australia; 3 Australian Institute of Health and Welfare, Canberra, Australia

**Keywords:** Life expectancy, Socioeconomic, Education, Inequalities, Mortality, Record linkage

## Abstract

**Background:**

Life expectancy in Australia is amongst the highest globally, but national estimates mask within-country inequalities. To monitor socioeconomic inequalities in health, many high-income countries routinely report life expectancy by education level. However in Australia, education-related gaps in life expectancy are not routinely reported because, until recently, the data required to produce these estimates have not been available. Using newly linked, whole-of-population data, we estimated education-related inequalities in adult life expectancy in Australia.

**Methods:**

Using data from 2016 Australian Census linked to 2016-17 Death Registrations, we estimated age-sex-education-specific mortality rates and used standard life table methodology to calculate life expectancy. For men and women separately, we estimated absolute (in years) and relative (ratios) differences in life expectancy at ages 25, 45, 65 and 85 years according to education level (measured in five categories, from university qualification [highest] to no formal qualifications [lowest]).

**Results:**

Data came from 14,565,910 Australian residents aged 25 years and older. At each age, those with lower levels of education had lower life expectancies. For men, the gap (highest vs. lowest level of education) was 9.1 (95 %CI: 8.8, 9.4) years at age 25, 7.3 (7.1, 7.5) years at age 45, 4.9 (4.7, 5.1) years at age 65 and 1.9 (1.8, 2.1) years at age 85. For women, the gap was 5.5 (5.1, 5.9) years at age 25, 4.7 (4.4, 5.0) years at age 45, 3.3 (3.1, 3.5) years at 65 and 1.6 (1.4, 1.8) years at age 85. Relative differences (comparing highest education level with each of the other levels) were larger for men than women and increased with age, but overall, revealed a 10–25 % reduction in life expectancy for those with the lowest compared to the highest education level.

**Conclusions:**

Education-related inequalities in life expectancy from age 25 years in Australia are substantial, particularly for men. Those with the lowest education level have a life expectancy equivalent to the national average 15–20 years ago. These vast gaps indicate large potential for further gains in life expectancy at the national level and continuing opportunities to improve health equity.

**Supplementary Information:**

The online version contains supplementary material available at 10.1186/s12939-021-01513-3.

## Introduction

Life expectancy is a key metric for population health, and by this measure, Australia is one of the healthiest countries in the world [1]. This summary measure refers to the average number of years people are expected to live from a given age, based on current mortality rates [2]. International comparisons of national life expectancy are useful as they provide an indication of a country’s health progress and potential for health gain. Hence life expectancies are routinely reported and used for monitoring purposes [3]. 

National-level estimates of life expectancy, however useful, can mask stark inequalities within countries. Estimates of life expectancy gaps between groups within a country are also needed; these estimates are not only powerful measures of inequalities within a country, they also provide valuable information on the potential for health gain from improvements in health equity. If large within-country inequalities in life expectancy exist and substantial proportions of the population are among those with lower life expectancy, addressing within-country inequalities would result in substantial improvements to national life expectancy.

One of the most prominent forms of health inequality relates to socioeconomic position, with well documented inequalities in health indicators [4, 5], including mortality [6, 7]. When measuring socioeconomic inequalities in health, the OECD recommends using education, measured at the individual-level: it is easily reported, generally stable in adulthood, less subject to reverse causality than other variables (e.g. income) and can be standardised across countries to enable international comparison [8, 9]. Many high-income countries routinely report within-country estimates of life expectancy according to education [3]. These estimates provide valuable international benchmarks of inequality for monitoring and policy purposes [10, 11].

However, Australian estimates of life expectancy according to education level have not been available, due to a lack of suitable data. Instead, estimates of inequality have been routinely based on area-level measure of socioeconomic position. These measures are based on the collective socioeconomic characteristics of people living in an area [12]. Using these area-level measures, socioeconomic inequalities in life expectancy in Australia are well documented [10, 11]: in 2011, the estimated gap in life expectancy at birth between those living in the most and least disadvantaged areas was 5.7 years for males and 3.3 years for females [10].

Inequality estimates based on area-level measures of socioeconomic position have provided valuable information but have limitations. Importantly, they are known to underestimate inequalities because of the substantial variation in socioeconomic position within areas [13]. Furthermore, they cannot be standardised for international comparison making it difficult to gauge how Australia performs relative to other countries. Estimates based on education (measured at the individual-level) are required to overcome these limitations.

In this study, we use recently linked whole-of-population data to estimate inequalities in adult life expectancy in relation to education, for men and women in Australia.

## Methods

### Data sources and study population

We conducted secondary data analysis using de-identified 2016 Census of Population and Housing data linked to Death Registrations (2016-17), available through the Multi-Agency Data Integration Project (MADIP). The Australian Bureau of Statistics linked the data via the Person Linkage Spine (hereafter, the Spine), a person-level linkage key created by linking information from Medicare Enrolments, Social Security and Related Information, and Personal Income Tax, with virtually complete coverage of residents of Australia [14].

The scope of the 2016 Census was usual residents of Australia on 9 August 2016 [14] and the estimated person response rate was 94.8 % [15]. Death Registrations available as part of the MADIP included all deaths registered in Australia [14] and were complete until the end of August 2017.

Our study population included Australian residents aged at least 25 years (the age at which education level becomes relatively stable) at the time of the 2016 Census, or who would turn 25 years during the study period, with a census record linked to the Spine.

### Variables

We derived highest level of education from two self-reported census variables: highest year of secondary school completed and highest non-school qualification. Consistent with OECD recommendations and in line with previous Australian research describing mortality inequalities according to education level [7, 16], we created five mutually exclusive categories. These were: university qualification, irrespective of whether Year 12 was completed (highest education level); other post-secondary school qualification and completed Year 12; other post-secondary school qualification but did not complete Year 12; no post-secondary school qualification but completed Year 12; and, no post-secondary school qualification and did not complete Year 12 (lowest education level). Missing data on education level (6.2 %, n = 902,246) were imputed using single imputation by an ordered logistic model (see supplementary material for more information).

We obtained sex (male and female) from the Census, month and year of birth from the Spine, and month and year of death from Death Registrations. Details on how we derived day of birth and day of death (used to calculate exact age and follow-up time) are provided in supplementary material.

### Analysis

We estimated sex- and education-specific life expectancies (with 95 % confidence intervals) for adults at 20-year intervals, from age 25 to 85 years. We estimated life expectancy using standard life table procedures [2], using mortality rates estimated from the linked Census-Deaths Registrations data, estimated as follows.

For each sex- and education-group, we estimated mortality rates by 5-year age group, starting from 25 to 29 years through to 100 years and older. Given that previous research found that mortality rates estimated using the 2016 Census linked to Death Registrations (2016-17) available through the MADIP differed to those in the Australian population [6], we estimated mortality rates by applying education-specific rate ratios, derived from the Census linked to Death Registrations, to population mortality rates based on all deaths occurring in 2016 (see supplementary material). Individuals contributed person-years-at-risk from the day they entered the study, either on 10 August or when they turned 25 years, and were followed until day of death or the end of the study period on 31 August 2017, whichever occurred first. We accounted for birthdays over the study period and apportioned person-years by age. Time survived for individuals who died was the difference between date of death and date of entry into the study, aggregated by 5-year age group.

We estimated absolute inequalities in life expectancy as the difference, in years, between life expectancy for the highest education level and each lower education level. To estimate relative inequalities, we calculated life expectancy ratios of each education level relative to the highest education level. For reporting purposes, we focused on differences in life expectancy between those with the highest (university qualification) and lowest (no qualification) education level. We estimated 95 % confidence intervals for life expectancies using 1000 Monte Carlo simulations [17] and for absolute difference estimates using standard errors of each life expectancy estimate.

To facilitate comparison with OECD estimates, we also estimated education-related inequalities in life expectancy at ages 30 and 65 using a three-category measure of education: high education (university qualification, International Standard Classification of Education [ISCED levels 6–8]), intermediate education (secondary graduation with/without other non-tertiary qualifications, ISCED levels 3–5) and low education (no secondary school graduation or other qualification, ISCED levels 0–2).

We validated our estimates of life expectancy by conducting broad comparisons with official estimates of life expectancy at ages 25, 45, 65 and 85 years for men and women and in relation to an area-level measure of socioeconomic position (Socio Economic Indexes for Areas [SEIFA] Index of Relative Socio-economic Disadvantage [IRSD] quintiles) at age 65 years (see supplementary material).

Analyses were conducted through the ABS virtual DataLab using Stata 16 [18] and R Studio [19].

## Results

There were 16,129,252 census records for Australian residents aged 25 years and older during the follow-up period. After excluding records that did not link to the Spine (n = 1,562,623, 9.7 %) or that linked in error (n = 656, < 0.01 %), there were 14,565,910 persons. Among this population, 140,954 deaths occurred in the follow-up period (98 % of Death Registrations where age at death was ≥ 25 years linked to the Spine).

After imputation of missing level of education data, 26.8 % (n = 3,896,201) of the study population were classified as having a university qualification, 17.0 % (n = 2,474,557) as other post-secondary and Year 12, 15.2 % (n = 2,210,066) other post-secondary and no Year 12, 12.5 % (n = 1,822,483) no post-secondary and Year 12 and 28.6 % (4,162,603) no post-secondary and no Year 12. Proportions with higher levels of education decreased with age (see Supplementary Table 1).

### Validation of linked data source

Our estimates of life expectancy at ages 25, 45, 65 and 85 years derived from 2016 population mortality rates for men and women were greater than, but within 0.4 years of, official estimates for 2014-16 (Supplementary Table 2). Comparison of estimates of life expectancy at age 65 years by SEIFA IRSD quintile derived from our linked analysis file compared with official area-based estimates revealed small differences (ratio of the two estimates ranged from 0.98 to 1.05 for men and 1.00 to 1.03 for women, Supplementary Table 3).

### Inequalities in life expectancy

Australian men and women with higher education levels had higher life expectancies than those with lower education levels (Table 1 and Supplementary Table 4). Life expectancy at age 25 was 61.2 years (95 %CI: 61.0, 61.4) for men with university education compared with 52.1 years (51.9, 52.3) for men with no qualifications, a life expectancy gap of 9.1 (8.8, 9.4) years (Table 1). The life expectancy gap was 7.3 (7.1, 7.5) years at age 45, 4.9 (4.7, 5.1) years at age 65 and 1.9 (1.8, 2.1) years at age 85.

For women, life expectancy at age 25 was 63.6 years (63.4, 63.9) for those with the highest education level and 58.1 years (58.0, 58.3) for those with the lowest level, a gap in life expectancy of 5.5 (5.1, 5.9) years. The life expectancy gap was 4.7 (4.4, 5.0) years at age 45, 3.3 (3.1, 3.5) years at age 65 and 1.6 (1.4, 1.8) years at age 85.

Relative differences in life expectancy between the highest education level and all other levels of education were larger for men than for women (men with the lowest education level had a 15–25 % reduction in life expectancy; women a 10–20 % reduction). For men and women, relative differences were also larger with increasing age (relative differences at age 25 years were 15 % for men and 9 % for women, at age 85 years, relative differences were 25 % for men and 19 % for women).

**Table. 1:** Absolute and relative differences by education level in life expectancy at ages 25, 45, 65 and 85 years for Australian men and women, 2016

	Life expectancy for men (95% CI)	Difference in years (95% CI)	Ratio	Life expectancy for women (95% CI)	Difference in years (95% CI)	Ratio
**Life expectancy by education level**						
** At age 25 years**						
University qualification (highest)	61.2 (61.0, 61.4 )	0.0	1.00	63.6 (63.4, 63.9)	0.0	1.00
Other post-secondary & Yr12	59.5 (59.3, 59.7)	1.7 (1.4, 2.0)	0.97	63.3 (63.0, 63.5)	0.3 (-0.1, 0.7)	1.00
Other post-secondary, no Yr12	57.6 (57.4, 57.7)	3.6 (3.3, 3.9)	0.94	61.9 (61.6, 62.1)	1.7 (1.3, 2.1)	0.97
No post-secondary & Yr12	57.2 (56.9, 57.4)	4.0 (3.6, 4.4)	0.93	61.3 (61.1, 61.6 )	2.3 (1.9, 2.7)	0.96
No post-secondary, no Yr12 (lowest)	52.1 (51.9, 52.3)	9.1 (8.8, 9.4)	0.85	58.1 (58.0, 58.3)	5.5 (5.1, 5.9)	0.91
**At age 45 years**						
University qualification (highest)	41.6 (41.4, 41.8)	0.0	1.00	44.0 (43.7, 44.2)	0.0	1.00
Other post-secondary & Yr12	40.3 (40.1, 40.5)	1.3 (1.0, 1.6)	0.97	43.8 (43.5, 44.0)	0.2 (-0.2, 0.6)	1.00
Other post-secondary, no Yr12	38.6 (38.5, 38.8)	3.0 (2.7, 3.3)	0.93	42.7 (42.5, 42.9)	1.3 (0.9, 1.7)	0.97
No post-secondary & Yr12	38.3 (38.0, 38.5)	3.3 (2.9, 3.7)	0.92	42.0 (41.8, 42.2)	2.0 (1.6, 2.4)	0.95
No post-secondary, no Yr12 (lowest)	34.3 (34.2, 34.4)	7.3 (7.1, 7.5)	0.82	39.3 (39.2, 39.4)	4.7 (4.4, 5.0)	0.89
**At age 65 years**						
University qualification (highest)	22.9 (22.8, 23.1)	0.0	1.00	25.0 (24.8, 25.2)	0.0	1.00
Other post-secondary & Yr12	22.1 (22.0, 22.3)	0.8 (0.6, 1.0)	0.97	25.0 (24.8, 25.3)	0.0 (-0.4, 0.4)	1.00
Other post-secondary, no Yr12	20.9 (20.8, 21.0)	2.0 (1.8, 2.2)	0.91	24.2 (24.0, 24.4)	0.8 (0.5, 1.1)	0.97
No post-secondary & Yr12	20.7 (20.5, 20.9)	2.2 (1.9, 2.5)	0.90	23.6 (23.4, 23.8)	1.4 (1.1, 1.7)	0.95
No post-secondary, no Yr12 (lowest)	18.0 (17.9, 18.1)	4.9 (4.7, 5.1)	0.79	21.7 (21.6, 21.8)	3.3 (3.1, 3.5)	0.89
**At age 85 years**						
University qualification (highest)	7.64 (7.46, 7.83)	0.0	1.00	8.67 (8.46, 8.88)	0.0	1.00
Other post-secondary & Yr12	7.87 (7.68, 8.07)	-0.2 (-0.5, 0.0)	1.03	8.76 (8.52, 9.00)	-0.1 (-0.4, 0.2)	1.01
Other post-secondary, no Yr12	6.83 (6.72, 6.94)	0.8 (0.6, 1.0)	0.89	8.39 (8.21, 8.59)	0.3 (0.0, 0.6)	0.97
No post-secondary & Yr12	7.40 (7.21, 7.60)	0.2 (0.0, 0.5)	0.97	8.45 (8.29, 8.60)	0.2 (0.0, 0.5)	0.97
No post-secondary, no Yr12 (lowest)	5.70 (5.64, 5.76)	1.9 (1.8, 2.1)	0.75	7.06 (7.02, 7.11)	1.6 (1.4, 1.8)	0.81

Note: Difference in life expectancy, in years, is between each education group and the highest education level (university qualification). Life expectancy ratios is the ratio of life expectancy relative to the highest education level (university qualification). 95 % confidence intervals for life expectancy estimates were estimated using 1000 Monte Carlo simulations. Confidence intervals for absolute gaps in life expectancy were estimated using standard errors for each life expectancy estimate

## Discussion

In Australia, life expectancy at adult ages varies substantially according to level of education. Among men and women at all ages, life expectancy in 2016 was lowest among those with no educational qualification and rose with increasing education. Absolute gaps between those with a university level of education and those with no educational qualification were substantial, being as great as 9.1 years for men and 5.5 years for women aged 25 years, and decreased with age. These absolute gaps were equivalent to a 10–25 % reduction in life expectancy for those with the lowest compared to the highest education levels. These relative differences in life expectancy increased with age (for men and women) and, reflecting lower life expectancies, were generally larger for men than women.

Our study is the first to estimate education-related life expectancy in Australia from individual-level data with high population coverage (> 85 %) and census data linked prospectively to Death Registrations. As a result, our estimates are likely to be the most accurate estimates to date. We note that our estimates are larger than have been previously documented for Australia [3, 20]. Australian data presented in an OECD report, based on death registrations linked to census data for 2011-12, found education-related gaps in life expectancy at age 25 years of 6.6 years for men and 3.7 years for women [3]. However, these are likely to be underestimates as they were based on linked death registrations only (population denominators were sourced externally) and the weights used to correct for the relatively low linkage rate (80 %) did not account for differences in linkage rates by education [21], which are likely lower in lower education groups.

While we acknowledge that longer lives do not necessarily translate into healthier lives, findings from this study highlight substantial inequalities in achieving the high life expectancies reported at the national level for Australia. The life expectancy reported here at age 25 for men with the lowest education in 2016 is equivalent to that for all men at age 25 in 1998 (52.4 years), while for women the equivalent year is 2001 (58.3 years) [22], corresponding to a 15-20-year lag.

Stark inequalities in life expectancy have been reported for other population groups in Australia. This includes a gap of 8.6 years in life expectancy at birth between non-Indigenous and Indigenous males and 7.8 years for females [1], and 5.0 and 4.0 years for males and females, respectively, living in rural and remote areas compared with those in major cities [23]. As expected, education-related inequalities based on individual-level data are larger than inequalities based on area-level socioeconomic level (compare Table 1 [at age 65 years] and Supplementary Table 2; see also [4, 11]). This is consistent with the fact that area-level measures are known to underestimate socioeconomic inequalities because of substantial within-area variation in socioeconomic position [13]. The inequalities found by our study, together with those reported for other population groups in Australia, highlight the pressing need to address social inequalities and the ongoing opportunities for further improvements to population health.

A degree of caution is required when directly comparing inequality estimates across countries, given that life expectancy estimates and the associated gaps can reflect differences in methodologies and data quality. However, comparisons are likely to be useful to benchmark, broadly, the size of Australia’s education-related inequalities in mortality. Our finding that education-related gaps in life expectancy are considerable is consistent with international studies [3, 24–26]. The average absolute highest-lowest education gap in life expectancy at age 25 years for 25 OECD countries (including Australia) based on linked census-mortality data for 2015-17, was 7.4 years for men and 4.5 years for women [3]. While our estimates exceed these by 1–2 years, they are comparable to estimates for men in Belgium (9.9 years) and Slovenia (8.3 years) and for women in Sweden (5.0 years), Denmark (5.1 years) and Hungary (5.7 years) [3].

We found larger education-related differentials in life expectancy for men than women. This difference is typical of social differentials in general and has been shown to stem in part from the greater influence of men’s education and income on household resources, although this appears to be changing [27]. Our finding that life expectancy gaps are larger for men than women is consistent with previous research which has shown larger absolute and relative education-related inequalities in mortality rates for men compared with women, particularly from deaths caused by injuries and accidents and causes associated with smoking and alcohol use [6].

Given the available data, it was not possible to examine reasons for the observed gaps in life expectancy. Previous research indicates mortality inequalities are likely to reflect unfair differences in resources that promote and protect good health, including money, knowledge, power and social connections [28, 29]. Consistent with this, Australian research has shown that socioeconomic inequalities are evident in behaviour-related risk factors (e.g. smoking, physical activity) [30], health care [31] and health conditions [13]. Previous Australian research found the largest absolute education-related inequalities in mortality were due to chronic diseases amenable to prevention, including lung cancer, ischaemic heart disease and chronic lower respiratory disease [6]. These findings suggest that in addition to addressing upstream social, economic and cultural determinants of health [32], the health system will play an important role in addressing socioeconomic inequalities in life expectancy and realising further population health gains.

Ongoing monitoring of within-country inequalities in life expectancy will be critical to assessing Australia’s progress towards achieving reductions in inequalities in life expectancy [33]. However, even within countries, differences in data collection and linkage quality over time can limit ongoing monitoring and the ability to conduct historical assessment of the impact of policies. Linked data from the MADIP, assuming it is routinely updated and that linkage quality remains high, can facilitate ongoing monitoring of inequalities. Although efforts will need to be made to ensure these data are suitable for comparisons over time.

## Strengths and limitations

In this record linkage study, life expectancy estimates were based on data with high coverage of the Australian population and virtually complete ascertainment of deaths. Education-specific mortality rates were estimated by applying education-specific rate ratios to mortality rates from the full population. This method assumes that the rate ratios estimated from the analysis file are unbiased and can be generalised to the full population. Although we had virtually complete (98 %) ascertainment of deaths, a previous study using these data found evidence that among young women, socioeconomic inequalities in mortality rates were underestimated [6]. Given this, it is likely that our estimates also underestimate the true education-related gaps in life expectancy for women. Finally, estimates presented here should be interpreted as summaries of inequalities in life expectancy, given that the meaning of education (and its relationship to health) has cohort effects and that our life expectancy estimates are based on a hypothetical population ageing through mortality rates observed at one point in time.

## Conclusions

In Australia, education-related gaps in life expectancy are considerable, such that those with the lowest level of education in 2016 had a life expectancy equivalent to the national average 15–20 years prior. They are larger than have been documented in many other OECD countries and highlight the substantial potential for further improvements in population health if socioeconomic inequalities in health were addressed.

## Supplementary Information

**Additional file 1:**

## Data Availability

Data part of the Multi-Agency Data Integration Project are available for approved projects to approved government and non-government users. https://www.abs.gov.au/websitedbs/D3310114.nsf/home/Statistical+Data+Integration+-+MADIP.

## Declarations

**Ethics approval**.

Ethics approval for this study was granted by the Australian National University Human Research Ethics Committee (reference 2016/666).
